# Differential CFTR-Interactome Proximity Labeling Procedures Identify Enrichment in Multiple SLC Transporters

**DOI:** 10.3390/ijms23168937

**Published:** 2022-08-11

**Authors:** Benoît Chevalier, Nesrine Baatallah, Matthieu Najm, Solène Castanier, Vincent Jung, Iwona Pranke, Anita Golec, Véronique Stoven, Stefano Marullo, Fabrice Antigny, Ida Chiara Guerrera, Isabelle Sermet-Gaudelus, Aleksander Edelman, Alexandre Hinzpeter

**Affiliations:** 1 INSERM, U1151, Institut Necker-Enfants Malades, 75015 Paris, France; 2 CNRS UMR 8253, Université Paris Cité, 75015 Paris, France; 3 Center for Computational Biology, Mines Paris-PSL, PSL Research University, 75006 Paris, France; 4 Institut Curie, 75248 Paris, France; 5 INSERM U900, 75428 Paris, France; 6 INSERM US24/CNRS UAR3633, Proteomic Platform Necker, Universit Paris Cité—Federative Research Structure Necker, 75015 Paris, France; 7 Institut Cochin, Université Paris Cité, INSERM, U1016, CNRS UMR 8104, 75014 Paris, France; 8 Faculté de Médecine, Université Paris-Saclay, 94210 Le Kremlin-Bicêtre, France; 9 INSERM UMR_S 999, Hôpital Marie Lannelongue, 92350 Le Plessis-Robinson, France; 10 Centre de Référence Maladies Rares Mucoviscidose et Maladies de CFTR, Hôpital Necker Enfants Malades, European Reference National (ERN) Lung Center, 75015 Paris, France

**Keywords:** proximity labeling, cystic fibrosis, CFTR, SLC transporters, KCNK3, interactome

## Abstract

Proteins interacting with CFTR and its mutants have been intensively studied using different experimental approaches. These studies provided information on the cellular processes leading to proper protein folding, routing to the plasma membrane, recycling, activation and degradation. Recently, new approaches have been developed based on the proximity labeling of protein partners or proteins in close vicinity and their subsequent identification by mass spectrometry. In this study, we evaluated TurboID- and APEX2-based proximity labeling of WT CFTR and compared the obtained data to those reported in databases. The CFTR-WT interactome was then compared to that of two CFTR (G551D and W1282X) mutants and the structurally unrelated potassium channel KCNK3. The two proximity labeling approaches identified both known and additional CFTR protein partners, including multiple SLC transporters. Proximity labeling approaches provided a more comprehensive picture of the CFTR interactome and improved our knowledge of the CFTR environment.

## 1. Introduction

Cystic fibrosis (CF), the most common monogenic life-threatening disease, is caused by mutations of the cystic fibrosis transmembrane conductance regulator (CFTR) gene [1], which encodes for a chloride channel located at the apical membrane of respiratory epithelial cells [2].

CFTR protein partners have been intensively studied, enabling a better understanding of the cellular processes leading to proper protein folding, its transport to the plasma membrane, recycling and degradation. Numerous protein partners implicated in these different steps have been identified (reviewed in this special edition [3]). They have often been identified based on the comparison between WT-CFTR and the CFTR-F508del mutant [4,5], the most frequent CF-causing mutation, or other misfolded mutants [6,7]. These interactions occur in different cellular compartments, which correspond to different steps in the CFTR biogenesis route. The first set of protein partners locates within the endoplasmic reticulum and is mainly implicated in CFTR synthesis and folding (reviewed in Refs. [8,9]). Some of them are implicated in the ER quality control (ERQC) of CFTR, recognizing misfolded channels and targeting them to proteasomal degradation. ERQC includes different checkpoints involving both chaperones, e.g., calnexin, calreticulin, Hsp70 and their co-chaperones, and specific motifs located on CFTR, such as RXR motifs implicated in ER retention and a diacidic exit code (DAD), which is involved in the recruitment of CFTR cargo into vesicles budding form ER exit sites [10]. It has been proposed that proper folding of CFTR reduces the accessibility to RXR motifs, favoring the ER exit of correctly folded channels [10,11,12]. After complex glycosylation in the Golgi apparatus, CFTR is exported to the plasma membrane where it associates with different types of proteins, such as membrane anchoring proteins, which link the channel to the cytoskeleton, or endosomal proteins implicated in the vesicular recycling of CFTR [3,13]. As in the ER, a peripheral quality control system monitors protein quality and targets altered channels to lysosomal degradation [14,15]. Finally, once at the cell surface, CFTR channel activity is mainly regulated by phosphorylation of its regulatory domain [16]. Several kinases participate in this regulation, mainly PKA [17,18] and, to a lesser extent, PKC [19,20] and tyrosine kinases [21]. Recently, Mihalyi et al. showed that CFTR association with PKA initiated conformational changes leading to channel activation, the phosphorylation of specific residues being necessary to maintain the effect over time [22]. Similarly, a specific protein–protein interaction between CFTR and WNK1 was recently shown to modulate channel selectivity toward bicarbonate versus chloride ions [23], an effect independent of the kinase activity of WNK1.

CFTR has also been shown to modulate the cell surface activity of other channels and transporters, such as ENaC [24], ORCC [25], SLC26A9 [26,27,28], SLC26A3 [29] or SLC26A6 [30]. Co-activation of CFTR and SLC26 transporters was associated with direct interactions between the STAS domain of SLC26 transporters and the R domain of CFTR [27,29]. The CFTR C-terminal PDZ domain also plays a key role in protein–protein interactions at the plasma membrane, anchoring CFTR to the cytoskeleton and enabling interactions with other PDZ containing proteins via PDZ-binding proteins, such as NHERF1 [9].

The CFTR interactome appears to be location specific and highly dynamic, affecting several steps in CFTR biogenesis, turnover and activity. Several approaches have been used to identify CFTR partners, such as yeast two-hybrid screens and CFTR immunoprecipitation coupled to mass spectrometry. These strategies have provided detailed CFTR interactome maps and are constantly improving. While yeast two-hybrid screens remain challenging for transmembrane proteins. These strategies have provided detailed CFTR interactome maps and are constantly improving. While, yeast two-hybrid screens remain challenging for transmembrane proteins leading to the use of CFTR fragments as baits, technological advances now enable to screen full-length CFTR in mammalian cells [31] Immunoprecipitation approaches require cell lysis with detergents that may modify the interactome compared to interactions taking place in a living cell [32,33,34,35]. Furthermore, the specificity of the obtained interactomes depends on the availability and specificity of antibodies as well as the experimental conditions.

To address these limiting aspects, new techniques have recently been developed to label protein partners in a native environment. These include, among others, BioID, TurboID and APEX2 proximity labeling enzymes [32,33,34,35], which are fused to the protein of interest. BioID is an Escherichia coli biotin ligase, which biotinylates proteins on lysine residues in a radius of approximately 10 nm. While the low activity of BioID usually requires between 18 h to 24 h of labeling, the sequence optimization of the enzyme resulted in a mutant ligase, called TurboID, characterized by enhanced enzymatic activity, reducing the labeling time to 10 min [33]. Another strategy is based on APEX2, a peroxidase allowing the labeling of protein partner electron-rich amino acid residues with a biotin derivative (Biotin-Phenol) at a spatial resolution of approximately 20 nm [33,35]. The labeling reaction is induced by adding H_2_O_2_ for a short period of time in living cells (1 min), providing a snapshot of the proximal interactome.

In this study, we explored and compared CFTR interactomes using three proximity labeling approaches, i.e., APEX2, BioID and TurboID. Experiments were performed in transiently transfected HEK293 cells to achieve high expression levels of fusion proteins and facilitate mass spectrometry identification.

## 2. Results

### 2.1. Proximity Labeling Approaches

**Figure 1:**
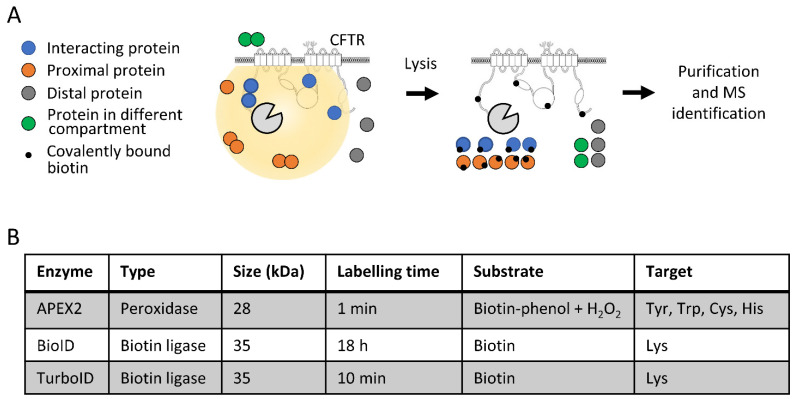
Characterization of fusion proteins. (**A**) Schematic representation of the proximity labeling strategy. Labeling enzymes were fused to the N-terminus of CFTR to biotin tag interacting and proximal proteins, while distal proteins and proteins separated by a membrane were not tagged. Labeled proteins were purified with streptavidin-coated beads and identified with mass spectrometry. (**B**) Characteristics of the fused enzymes used in the study, indicating the type of activity, the size, the recommended labeling time, the substrate used and the targeted amino acid.

The coding sequences of BioID, TurboID and APEX2 were subcloned upstream of that coding for CFTR to generate fusion proteins containing the enzymes at the N-terminus of CFTR. A linker region consisting of five glycine-serine repeats (GS5) motifs was introduced between BioID/APEX2/TurboID and CFTR to improve flexibility and to decrease CFTR near-end crowding. The activity of these fusion proteins enabled the labeling of proteins interacting with CFTR or proximal proteins within a radius of 10–20 nm, while distal proteins or proteins separated by a membrane were not labeled (Figure 1A). The covalent labeling of interacting and proximal proteins with biotin was not affected by cell lysis procedures under denaturing conditions, such as with a RIPA buffer. Biotinylated proteins were purified using streptavidin-coated beads, washed and eluted in denaturing Laemmli buffer [35]. Samples were then digested and analyzed by mass spectrometry for protein identification (Figure 1A). While the overall procedure was similar for the three fusion proteins, some specificities exist, such as the length of labeling times (from 1 min to 18 h) and the targeted amino acids (Lys versus Tyr/Trp/Cys, His) (Figure 1B). The peroxidase activity of APEX2 requires biotin-phenol as a substrate and H2O2 addition to activate the enzyme, while BioID and TurboID require a biotin pulse for labeling (Figure 1B).

### 2.2. Characterization of Fusion Proteins

**Figure 2:**
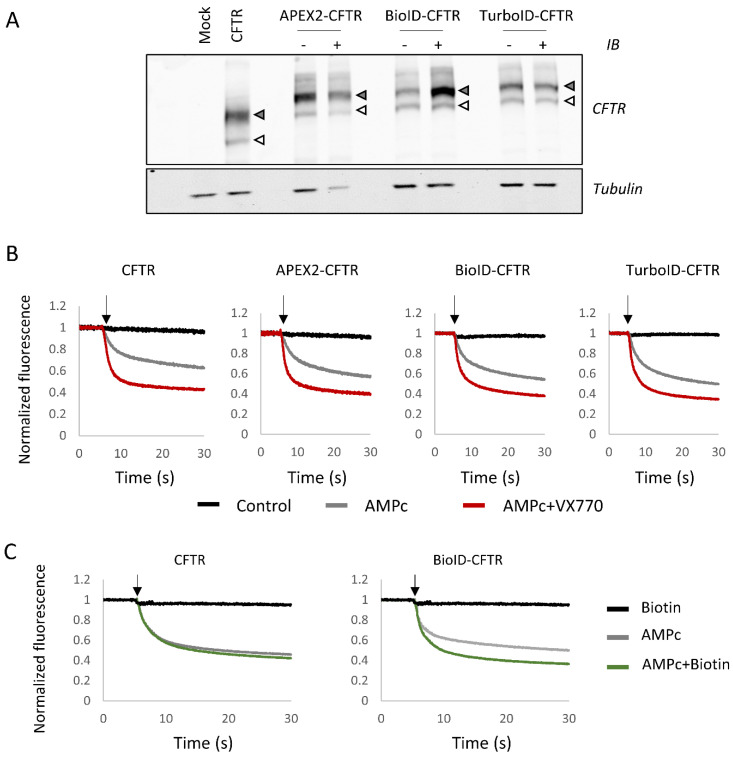
Characterization of CFTR fusion proteins. (**A**) HEK293 cells are transfected with either an empty vector (Mock), CFTR without fusion (CFTR) or CFTR fused with proximity labeling enzymes (APEX2-CFTR, BioID-CFTR and TurboID-CFTR). For CFTR fusions with proximity labeling enzymes, cells are either untreated (indicated as −) or treated under biotinylation-inducing conditions (indicated as +) (see Materials and Methods). The upper panel corresponds to the detection of CFTR with band B (white arrow head) and band C (gray arrow head). In the bottom panel, Tubulin was used to assess equal loading. (**B**,**C**) Halide-sensitive YFP assay of fusion proteins measured in HEK293 transfected cells. The arrow indicates the time point of PBS NaI injection. Measures were performed in control conditions (Control, black) after a 30 min incubation with cpt-AMPc/IBMX to activate CFTR (AMPc, gray) or with cpt-AMPC/IBMX and 1 µM VX-770 (AMPc + VX-770, red). Cells were also incubated with 10 mM biotin for 18 h (**C**) prior to measurements (Biotin, black and AMPc + Biotin, green).

N-terminal fusions of BioID, TurboID and APEX2 with CFTR WT were first analyzed by Western blot. Untagged CFTR expressed in HEK293 cells was detected under the form of two bands, one corresponding to core-glycosylated CFTR (band B), the second, more diffuse band corresponding to fully glycosylated CFTR (band C). Fusion proteins showed the same pattern and intensity, with a size shift increase of approximately 30 kDa compared to WT CFTR due to the fusion of BioID, TurboID or APEX2 (Figure 2A). In addition to their conserved maturation pattern, fusion proteins were active, as shown by a halide sensitive fluorescent assay following cAMP stimulation, in the presence of the VX-770 potentiator (Figure 2B). These results are consistent with previous studies, indicating that the fusion of a GFP tag to the N-terminus of CFTR preserves the functional CFTR chloride channels [36]. As BioID labeling requires a much longer incubation period (18–24 h), the activity of BioID-CFTR fusion protein was measured after this longer biotin labeling. The results revealed enhanced channel activity (Figure 2C), concomitant with greater amounts of fully glycosylated BioID-CFTR in the Western blot analysis (Figure 2A). These results indicate that biotinylation of CFTR or CFTR partners enhanced channel stability at this prolonged time point.

### 2.3. Mass Spectrometry Identification

**Figure 3:**
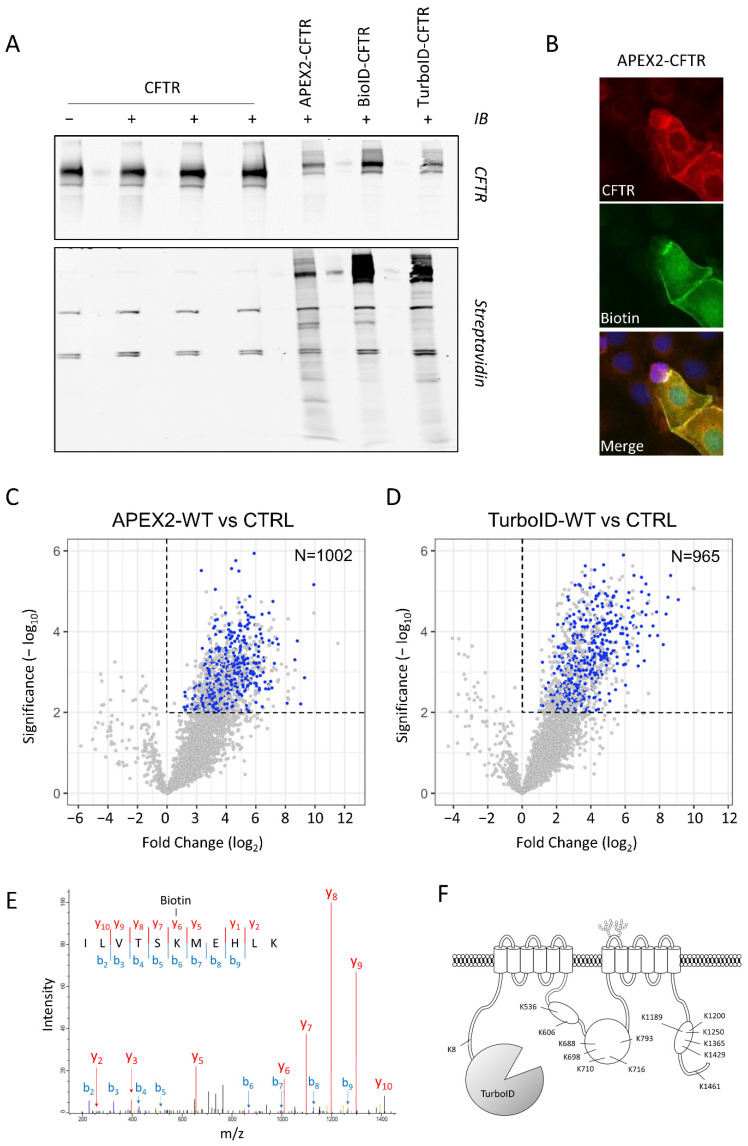
MS identification of CFTR partners. (**A**) Western blot analysis of CFTR fusion proteins and biotinylated proteins. The upper inlay was probed with CFTR antibody, while the lower was probed with streptavidin. Each labeling procedure was performed on both untagged and tagged CFTR, in the same order. (**B**) Localization of APEX2-CFTR transfected CFBE cells. CFTR was identified using CFTR antibody 24.1, biotinylated proteins using streptavidin-Alexa488 and nuclei using Hoechst dye. (**C**,**D**) Volcano plots of APEX2-CFTR (C, *n* = 3 replicates) and TurboID-CFTR (D, *n* = 4 replicates) versus matched non-transfected HEK293 cells (*n* = 3 and *n* = 4 replicates). APEX2-CFTR identified a total of 3088 proteins and TurboID-CFTR a total of 3054 proteins, of which 1002 and 965, respectively, are enriched compared to non-transfected controls. Blue dots indicate 433 proteins identified in both sets (Student’s *t*-test; *p*-value < 0.01). (**E**) Fragmentation mass spectrum of CFTR peptide 1184–1199 aa with biotinylation located on K1189 (one example of the 14 biotinylated peptides found for CFTR). (**F**) Position of the 14 biotinylated CFTR lysines identified in the TurboID-CFTR samples.

The labeling capacity of the fusion proteins was assessed in Western blots using fluorescent streptavidin. Biotinylation by APEX2 was initiated in living cells pre-incubated with Biotin-Phenol by the addition of H2O2 in the cell media for 1 min, while BioID and TurboID labeling required a biotin pulse of 18–24 h and 10 min, respectively. Upon the activation of APEX2, BioID or TurboID, a smear was visible, corresponding to CFTR partners that were biotin labeled in transiently transfected HEK293 cells (Figure 3A). The overall biotinylation intensity was similar between the different conditions, with notably higher labeling of CFTR with BioID and TurboID compared to APEX2 and some differences observed in the patterns (Figure 3A). Immunohistochemistry showed that the fusion protein was enriched at the cell surface, where the highest level of biotinylation was visible (Figure 3B). The diffusion of some biotinylated proteins within the cytoplasm reflected most probably the mobility of the proteins within the cell.

CFTR protein partners were identified using both APEX2 and TurboID labeling procedures (see Materials and Methods). After labeling, cells were lysed and the biotinylated proteins purified using streptavidin-coated beads. Mass spectrometry analysis of purified biotinylated preys identified more than one peptide in 3088 proteins for the APEX2 and in 3054 proteins for the TurboID procedure (Supplementary Table). Among them, 1002 and 965, respectively, were enriched in positive samples as compared to the non-transfected negative control (Student’s *t*-test, *p*-value < 0.01), and 433 proteins were found enriched with both procedures (Figure 3C,D, blue dots).

Moreover, CFTR was found to be biotinylated as well, and multiple peptides carried the modification (Figure 3E), confirming the specificity of the procedure and validating the approach. Specifically, biotinylated lysine residues were located in the different cytoplasmic regions of CFTR: N-terminus, NBD1, R-domain and NBD2 (Figure 3F), without any labeling within or across the membranes.

### 2.4. Analysis of Proximal Datasets and Comparison to Biogrid

**Figure 4:**
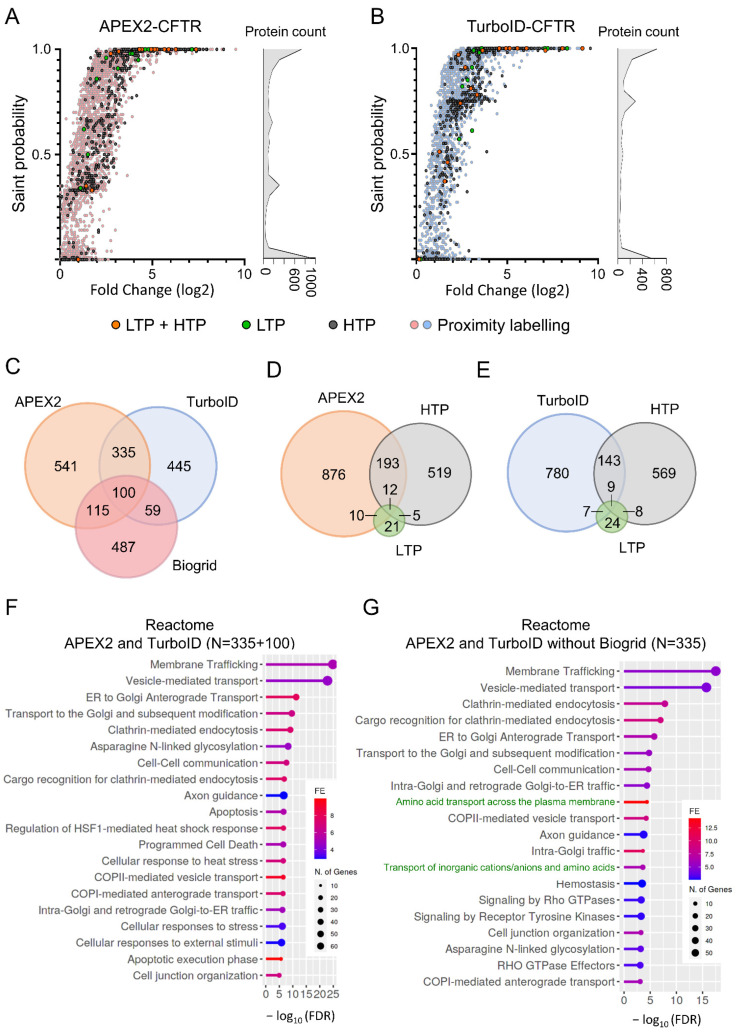
Analysis of proximal datasets and comparison to Biogrid. (**A**,**B**). The computational tool SAINT assigns confidence scores to protein–protein interaction. Analysis using SAINT of the datasets obtained with the APEX2 ((**A**), *n* = 3 replicates, 4490 total proteins) or the TurboID ((**B**), *n* = 4, 3356 total proteins) procedure. The X axis indicates the fold change of intensities for each individual interaction compared to control purifications. CFTR partners also referenced in Biogrid database, either from low-throughput (LTP, in green), high-throughput (HTP, in dark gray) or both (LTP + HTP, in orange) studies are also indicated. (**C**) Venn diagram performed between APEX2 and TurboID datasets. A FDR < 1% was used to identify high confident proximal partners (*n* = 1091 for APEX2 and *n* = 939 TurboID). (**D**,**E**) Venn diagram performed between APEX2 (**D**) and TurboID (**E**) datasets and CFTR partners referenced in Biogrid database, either from low-throughput (LTP) or high-throughput (HTP) studies. (**F**,**G**) Reactome enrichment analysis of the 435 proteins identified with both APEX2 and TurboID procedures as high confident proximal partners (FDR < 1%) (**F**) or 335 specifically detected using both APEX2 and TurboID but not referenced in Biogrid with, in green, terms associated with solute transport (**G**).

The two total datasets were then analyzed using the Significance Analysis of INTeractome (SAINT) probabilistic scoring tool [37] to identify the high confident proximal partners (FDR < 1%) for APEX2 (*n* = 1091) and TurboID (*n* = 939) (Figure 4A,B). The comparison of these groups of high confident CFTR proximal partners identified 435 common proteins, representing 39.7% of the APEX2 group and 46.3% of the TurboID group (Figure 4C). We then compared the datasets to the Biogrid database (Figure 4D,E), which categorizes interactants reported in the literature from low-throughput studies (LTPs, in green) on specific CFTR interactants and from high-throughput experiments (HTPs, in dark gray) corresponding to immunoprecipitation studies followed by mass spectrometry [4,38]. The APEX2 and TurboID procedures identified a similar proportion of CFTR interactants found by LTPs and HTPs. Interestingly, some interactants from LTPs that were not identified previously by HTPs were detected by proximity labeling—10/48 proteins for APEX2 and 7/48 proteins for TurboID (Figure 4D,E). Of note, a large number of partners reported in the LTPs (Figure 4A,B, in green) and LTP + HTP (Figure 4A,B, in orange) datasets were part of the high confident proximal partners (FDR < 1%), while proteins in the HTP dataset showed more dispersion (Figure 4A,B, in dark gray).

We then performed a gene ontology (GO) enrichment analysis of the 435 proteins identified in both the APEX2 and TurboID datasets to delineate the cellular functions and biological processes involved in CFTR biogenesis function or regulation. We observed enrichments with terms associated with protein localization and intracellular vesicular transport of CFTR (Figure 4F and Supplementary Figure S1A,B). One of the strongest enrichments corresponded to SNAP receptor activity (Supplementary Figure S1B,C), which is involved in membrane fusion during vesicular transport. Some of these SNAREs were reported to interact with CFTR and to impact CFTR biogenesis [39]. Proteins associated with small GTPases were also highly enriched, including regulators of the Rab small GTPases (RAB11FIP1 and RAB3GAP2) involved in vesicular transport and effectors of the RhoA signaling pathway (ROCK1/2) (Supplementary Figure S1B,C). This finding is in agreement with previous studies reporting cross talks between the CFTR function or processing and the RhoA/ROCK pathway, including Refs. [40,41,42]. APEX2 and TurboID proximity labeling also enabled the identification of interaction partners of CFTR folding, including chaperones and proteins involved in the ubiquitination process (Supplementary Figure S1B,C).

We finally evaluated whether these approaches could reveal novel biological signaling pathways or CFTR functions. We therefore searched for enrichments within a set of proteins common to both APEX2 and TurboID but not yet described as interacting with CFTR in the Biogrid database (N = 335 proteins). The Reactome database identified enrichment for biological pathways associated with CFTR trafficking, as described with the previous set (Figure 4F,G). Multiple terms associated with solute transport were also found to be enriched, each of them containing SLC transporters (Figure 4G, in green). Detection of SLC transporters was more efficient using APEX2 (*n* = 18) and especially TurboID (*n* = 34) as compared to methods referenced in Biogrid (*n* = 11) (Supplementary Figure S1D). This is also true for the global detection of transmembrane proteins, as TurboID detected almost twice as many (30.6%) transmembrane proteins compared to APEX2 (16.2%) and the Biogrid set (16.7%) (Supplementary Figure S1E).

### 2.5. Comparison of CFTR-WT Versus Mutant CFTR-G551D and -W1282X

In order to evaluate the impact of mutations on the dynamics of the CFTR network, we compared the interactome of CFTR-WT with mutant CFTR-G551D and CFTR-W1282X. These two mutations induce distinct functional defects. CFTR-G551D alters channel gating by affecting ATP binding and/or NBD dimerization while preserving the global architecture and localization of the channel. Proximity labeling of CFTR-G551D showed important similarities with CFTR-WT. A comparison of proteins identified in each replicate showed few changes in the proximal interactome obtained with both APEX2 (22 out of 1966 proteins) and TurboID (9 out of 1654 proteins) (Student’s *t*-test, *p*-value < 0.1, Supplementary Figure S2A,B). However, the gene set enrichment analysis (GSEA) indicated an enrichment for some GO terms, indicative of enhanced proximity of CFTR to the endoplasmic reticulum membrane (APEX2 dataset) or other GO terms associated with the plasma membrane as well as the actin cytoskeleton (TurboID dataset). Nonetheless, among the identified interacting proteins, no proximal partners appeared lost or gained for G551D (Supplementary Figure S2C,D), which suggests that the CFTR interactome is minimally perturbed for this mutant.

**Figure 5:**
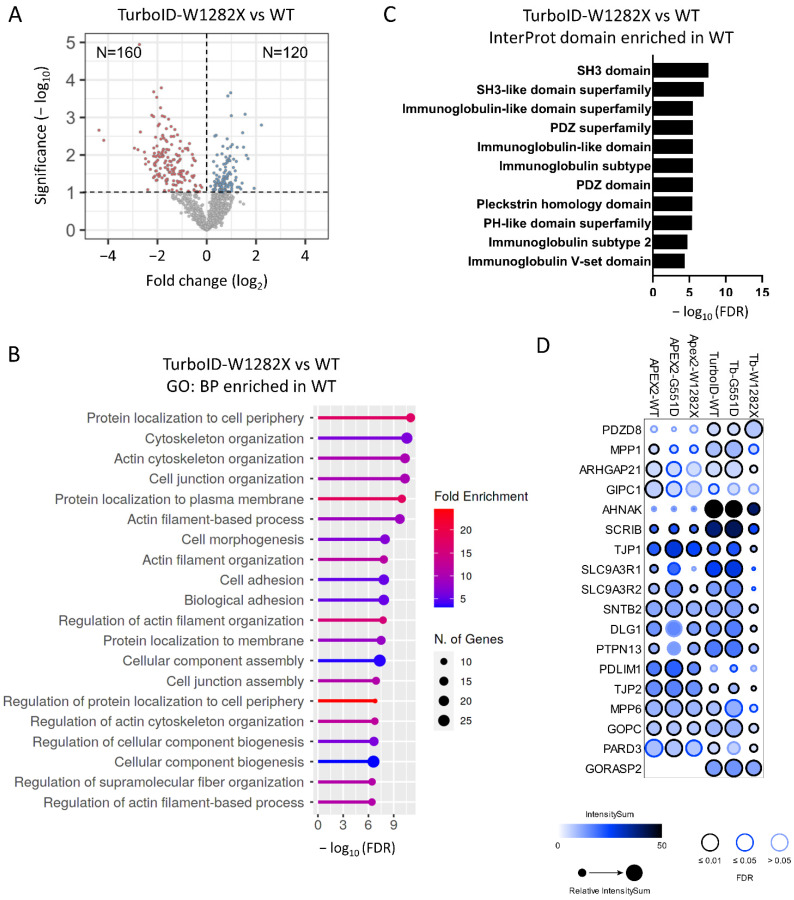
Analysis of TurboID-CFTR-W1282X proximal dataset. (**A**) Volcano plot of TurboID-CFTR-W1282X versus TurboID-CFTR-WT transfected HEK293 cells (*n* = 4 replicates, 1654 total proteins). Blue dots indicate proteins identified as enriched in the W1282X sample (Student’s *t*-test, *p*-value < 0.1, *n* = 120) and red dots in the WT sample (Student’s *t*-test, *p*-value < 0.1, *n* = 160). (**B**) GO enrichment terms identified in TurboID-CFTR-W1282X. (**C**) Enrichment analysis of Pfam domain in proteins enriched in TurboID-CFTR-WT compared to TurboID-CFTR-W1282X. (**D**) PDZ domain proteins enriched in TurboID-CFTR high confident proximal partners (FDR < 1%), shown as dot plots with ProHits-viz [43]. The color of each circle represents the intensity; the circle size indicates the relative value of the intensity across APEX2 and TurboID and confidence in the measurement via colored edge.

The W1282X mutation truncates part of NBD2 and the end C-terminus, which contains the PDZ domain of the protein, leading to both protein instability and abrogation of channel function. The differential interactome between WT and W1282X showed several differences between the APEX2 and TurboID assays, with 101 and 280 proteins enriched (Student’s *t*-test, *p*-value < 0.1) (Supplementary Figures S2E and Figure 5A). The gene enrichment analysis for APEX2 indicated an enrichment of W1282X with mitochondria-associated terms (Supplementary Figure S2F). For TurboID, the analysis indicated enrichment of proteins related to the misfolding protein response (Supplementary Figure S3A,B) in addition to the expected significant loss of terms related to the plasma membrane (Figure 5B). Additionally, the Interprot domain enrichment analysis showed the expected drastic drop in the number of proteins with a PDZ domain (Figure 5C). Among these proximal CFTR interacting partners, scaffolding proteins, such as SLC9A3R1 and SLC9A3R2 (NHERF1 and NHERF2), were strongly reduced or lost with W1282X (Figure 5D). This loss of interaction was particularly observed with the TurboID approach and, to a lesser extent, with the APEX2 approach (Figure 5D).

### 2.6. Comparison of TurboID-CFTR and KCNK3-TurboID Interactomes

**Figure 6:**
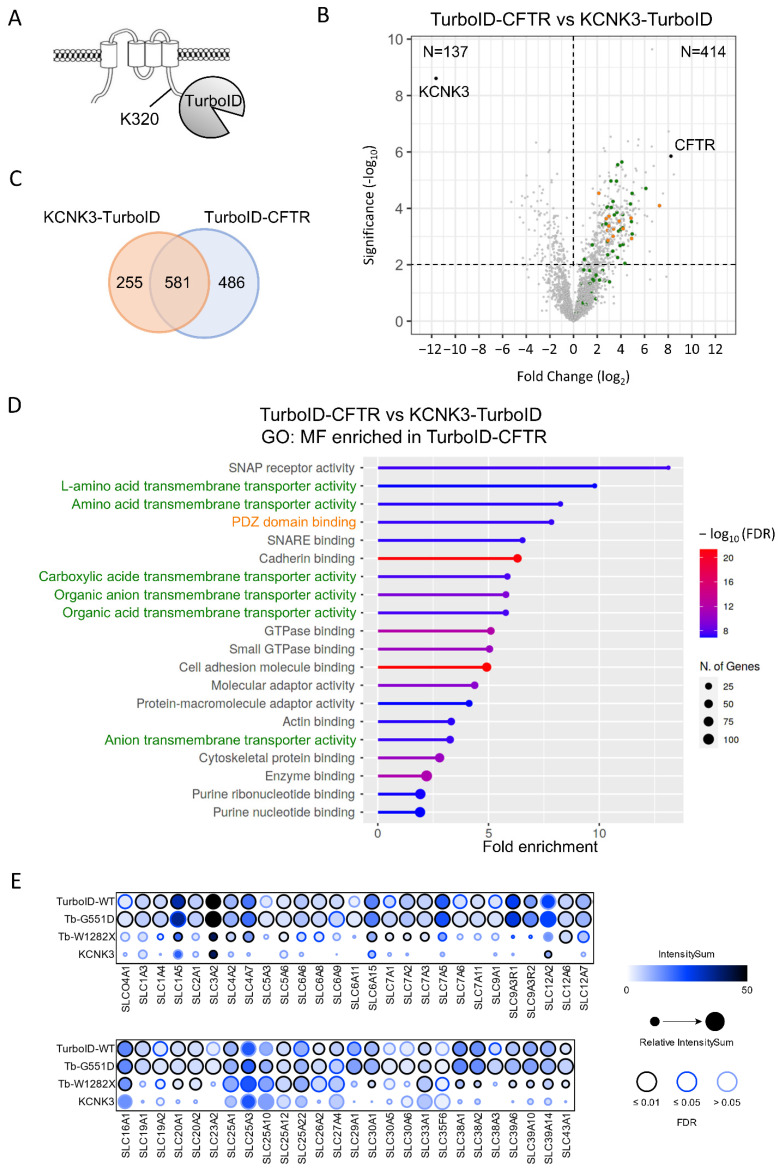
Comparison of TurboID-CFTR and KCNK3-TurboID proximity labeling. (**A**) Schematic representation of KCNK3-TurboID topology. (**B**) Volcano of proteins identified in TurboID-CFTR and KCNK3-TurboID conditions (*n* = 4 and *n* = 5 replicates, 3041 total proteins). Proteins enriched in the CFTR sample (Student’s *t*-test, *p*-value < 0.01, *n* = 414) are in top right panel, and proteins enriched in KCNK3 sample (Student’s *t*-test, *p*-value < 0.1, *n* = 137) are in top left panel. Green dots indicate proteins identified as enriched in TurboID-CFTR samples associated with SLC transporters in the GO: Molecular Function (**D**) and in orange enriched in PDZ/PDZ binding domain in the Interpro domain analysis (Supplementary Figure S4B). (**C**) Venn diagram performed on TurboID-CFTR and KCNK3-TurboID partners. (**D**) GO enrichment terms identified in TurboID-CFTR Molecular Function (GO: MF). (**E**) SLC members detected in at least one condition as high confident partners (FDR < 1%) are shown as dot plots with ProHits-viz [43]. The color of each circle represents the intensity; the circle size indicates the relative value of the intensity across APEX2 and TurboID and confidence in the measurement via colored edge.

In order to highlight the protein partners, which specifically and selectively bind to CFTR, we next compared the CFTR interactome with that of KCNK3. KCNK3 is a pH-dependent, voltage-insensitive, background outward potassium channel, structurally unrelated to CFTR but with similar biogenesis and final cell localization. It is formed by protomers of four transmembrane domains, which dimerize to form the pore (Figure 6A). A C-terminal KCNK3-TurboID fusion protein was generated, which maintained the same activity as the untagged channel in whole-cell patch-clamp recordings performed in transiently transfected HEK293 cells (data not shown). Proximity labeling was performed with KCNK3-TurboID in HEK293 cells, and, as observed for TurboID-CFTR, a biotinylated peptide was identified, corresponding to the modification of the amino acid residue K320 (Figure 6A). Comparison with the TurboID-CFTR dataset (Figure 6B,C) showed the presence of both common protein partners and proteins more specific to either CFTR or KCNK3 (Student’s *t*-test, *p*-value < 0.01). Pathway enrichment analysis showed that common partners were mainly involved in protein biogenesis with enriched terms associated with Golgi vesicle transport and intracellular protein transport (Supplementary Figure S4A). The analysis of proteins interacting specifically with CFTR indicated a strong enrichment in terms associated with the plasma membrane and proteins containing a PDZ binding domain (Figure 6B,D, orange, and Supplementary Figure S4B). However, the strongest enrichments corresponded with terms associated with the activity of transporters (Figure 6B,D, green), among which we found proteins belonging to three large families of transporters: SLC transporters (Figure 6E), ATP transporters (Supplementary Figure S4B) and ABC transporters (Supplementary Figure S4B). The 53 SLC proteins detected with CFTR were completely absent in the KCNK3 interactome and partially lost with CFTR-W1282X (Figure 6E). However, the effect was much smaller on ATP and ABC transporters (Supplementary Figure S4B), suggesting a close proximity of CFTR with multiple SLCs.

## 3. Discussion

Novel techniques based on proximity biotin labeling provide a snapshot of the CFTR environment with both direct binding partners and proximal non-interacting proteins.

Covalent biotin binding to specific amino acid residues can affect their post-transcriptional modification and/or their conformation. This could explain the results obtained with BioID-CFTR, where 18 h labeling led to the increase in both CFTR activity and concentration (Figure 2C). It is possible that biotinylation on specific lysine residues prevents their ubiquitination and, consequently, CFTR trafficking and degradation. CFTR ubiquitination on multiple lysine residues was reported by several teams [44,45]. Some ubiquitinated lysines were identified by mass spectrometry after TurboID assays (e.g., K536, K698, K710, K716, K793, K1250). The stabilization of both the bait and interacting partners upon biotinylation has been reported in other contexts [46]. This probably also occurs during the 10 min labeling with TurboID, possibly affecting CFTR behavior but to a lesser extent. CFTR biotinylation and/or stabilization could alter the interactome, preventing some interactions or inducing interactions that do not normally occur.

Both TurboID and APEX2 approaches identified multiple proteins associated with CFTR. In our study, APEX2 and TurboID identified a similar total number of proteins, and around 50% of the proteins were identified by the two methods. Label-free quantifications (LFQ) of proteins identified with both procedures were comparable and the CFTR protein intensity similar (Supplementary Figure S5A). Principal component analysis (PCA) of the APEX2 datasets showed experiment-dependent clustering, a feature not observed with the TurboID dataset (Supplementary Figure S5B) or when performing analysis between the CFTR mutants (Supplementary Figure S5C). Differences between the two approaches could be related to the reactivity of the Biotin-phenol, its diffusion radius or the usage of H_2_O_2_ (Figure 1B). It has been reported that different organelles with distinct pH, redox environments and endogenous nucleophile concentrations may influence the proximity ligation activity [34]. The subcellular distribution of the different datasets was explored using SubcellulaRVis, which calculates enrichment for 14 subcellular compartments (Supplementary Figure S6). Differences between APEX2 and TurboID showed that APEX2 had a stronger enrichment for proteins associated with the cytoplasm and the cytoskeleton, while TurboID showed higher enrichments for proteins associated with the endoplasmic reticulum and the Golgi apparatus. Few differences were observed between the two methods for plasma membrane proteins or intracellular vesicles. However, these observations cannot explain the totality of the differences between the two methods. Additionally, labeling is based on the covalent binding of biotin to specific amino acid residues, which may be more or less accessible under native conditions. Of note, the 10 min biotinylation pulse in the TurboID procedure can be prolonged to increase the number of proteins identified but can possibly affect protein synthesis or degradation and enhance non-specific background.

The CFTR interactome has been extensively studied by co-immunoprecipitation (co-IP) coupled with mass spectrometry [4]. Differences between biotin labeling approaches and co-IPs include the necessity to fuse the biotin ligase (or peroxidase) to the protein of interest. The fusion procedure can alter protein expression, folding and function, parameters that need to be evaluated. Even if tagged and untagged CFTR showed similar maturation and function (Figure 2A), the accessibility to the N-terminal region could be affected due to steric hindrances caused by the fused protein. In CFTR, the N-terminal region was shown to be engaged in interactions with Filamin A [47], syntaxins [39,48,49,50] and WNK1 [23]. Only around half of the partners identified by co-IP were also identified with TurboID or APEX2 approaches. A major difference between the two approaches is the labeling of both transient partners and non-interacting but proximal proteins, which might not be co-immunoprecipitated. Of note, around half of the identified proteins were unique to each method (547 out of 1095 for APEX2, 446 out of 941 for TurboID and 466 out of 729 for coIP). In the same line, the recently developed MaMTH-HTS method, which used full-length CFTR-WT as a bait to screen a library of around 10,000 ORF [31], only marginally overlapped with proximity labeling or co-IP. Another important issue is the cell type used. In this study, transiently transfected HEK293 cells were used to achieve high expression levels. It has to be kept in mind that overexpression can affect CFTR interactome and that partners specific to lung epithelial cells or pancreatic duct cells where CFTR is endogenously expressed may be absent in HEK293 cells. It appears that combining different approaches and cell types is necessary to obtain a full picture of CFTR interactome, to feed the databases and provide a better understanding of the CFTR environment.

Compared to co-IP (HTS dataset), proximity labeling (especially with TurboID) identified a greater proportion of transmembrane proteins, such as transporters, and more specifically, SLC transporters (Supplementary Figure S1D,E). This probably reflects both the difficulty in preserving membrane protein complexes using detergents in the co-IP procedure and the labeling of non-interacting but proximal proteins, which might not be co-immunoprecipitated. Functional co-regulation between CFTR and members of the SLC26 subfamily was found to involve a direct protein–protein interaction between the STAS domain of SLC26 transporters and the R domain of CFTR [27,29]. In this “special issue”, CFTR biogenesis and stability were shown to be affected by SLC26A9 expression levels [26]. As many of the SLC transporters identified (Figure 6E) do not contain a STAS domain, it can be speculated that other domains could be involved, which still need to be identified. Alternatively, proximity could be driven by localization in particular sub-cellular compartments or the sharing of common pathways during protein biogenesis. While structurally unrelated, it is plausible that these large transmembrane transporters require specific protein complexes for their proper folding and expression at the cell surface. Finally, both CFTR [51,52] and some SLCs [53,54] have been identified within and outside sphingolipid- and cholesterol-rich lipid nanodomains (or lipid rafts), raising the possibility of defining specific regions where these transporters are in close proximity. Taken together, the close proximity between CFTR and SLC transporters should be further explored.

Finally, the comparisons between WT and mutant CFTR showed that multiple interacting partners were nevertheless preserved. CFTR-G551D showed very few differences with CFTR-WT, consistent with a global preservation of the channel structure and localization. As reported in a previous study [55], some GO enrichments were identified, suggesting a higher affinity of G551D for the actin network compared to CFTR WT. Another report showed multiple differences between WT and G551D [7] not found here. These discrepancies will need further studies and could be linked, in part, to different cell types used, CFBE41o- [7] versus HEK293 in our study, or HeLa cells [55]. CFTR-W1282X, which lacks part of NBD2 and the C-terminus of the protein, showed more differences, including, as expected, the PDZ binding proteins and proteins associated with protein misfolding. Protein misfolding is consistent with the stabilizing effect of the corrector VX-445 observed on CFTR-W1282X upon inhibition of nonsense-mediated decay [56].

The importance of the identified proximal proteins to CFTR biogenesis and function needs to be functionally evaluated, as their labeling could only reflect common cellular processes and localization within the same cellular subdomain. When comparing CFTR and KCNK3 interactomes, many proteins were identified in both sets, revealing common biogenesis pathways and localization. Nonetheless, clear differences were observed for sets of proteins that were enriched in CFTR samples, such as PDZ containing proteins (KCNK3 lacks a PDZ binding domain) and SLC transporters. For the latter, CFTR was found to be in the vicinity of multiple SLC transporters, some of which have been found to be functionally co-regulated with CFTR [27,29] or influence CFTR biogenesis [26]. This result indicates that while undergoing the same biogenesis pathways and localizing in the same compartments, these two channels are associated preferentially with distinct protein sets. The comparison of interactomes from specific channels or channel families could enable identifying signatures associated with their localization, function or regulatory pathways.

In conclusion, our study provides evidence that the various approaches developed for interactomic studies can each identify unique proteins and therefore should be combined to obtain a more complete picture of the CFTR interactome.

## 4. Materials and Methods

### 4.1. Plasmid Constructs

CFTR fusion constructs were obtained by PCR assembly (NEBuilder HiFi DNA Assembly, NEB, Évry-Courcouronnes, France) in the expression vector pLenti-III digested with NheI and XbaI. The assembly was performed for each construct to the digested pLIII plasmid with 3 fragments: a fragment obtained by PCR amplification (Q5^®^ High-Fidelity, NEB, Évry-Courcouronnes, France) of proximity labeling enzymes (APEX2, BioID or TurboID), a synthetic fragment corresponding to the GS5 linker and the N-terminal part of CFTR (Eurofins Genomics, Les Ulis, France), a second fragment obtained by PCR amplification of CFTR-WT or its mutants (amplification of the BlpI site up to the CFTR stop codon). The template BioID plasmid was obtained from Morgan Gallazzini, (Institut Necker Enfants Malades Paris France), APEX2 from Jacques Camonis, (Institut Curie Paris France); V5-TurboID-NES_pcDNA3 and C1(1-29)-TurboID-V5_pCDNA3 were a gift from Alice Ting (Addgene plasmid #107173 and #107173).

All plasmids obtained were entirely sequenced (Eurofins Genomic, Les Ulis, France). KCNK3 were obtained by PCR assembly using as template C1(1-29)-TurboID-V5_pCDNA3.

### 4.2. Cell Culture and Transfection

HEK293 cells were purchased from ATCC and cultivated in DMEM medium supplemented with 10% fetal calf serum (Thermo Fisher Scientific, Illkirch-Graffenstaden, France). Cells were maintained at 37 °C, 5% CO_2_. For the functional assay, cells were co-transfected with equal amount of halide-sensitive YFP and CFTR plasmids using Turbofect (Thermo Fisher Scientific, Illkirch-Graffenstaden, France). For Western blot analysis and mass spectrometry analysis, cells were transfected with CFTR plasmids using Lipofectamine 3000, following instructions (Thermo Fisher Scientific, Illkirch-Graffenstaden, France).

### 4.3. Western Blot Analysis

The transfected cells were lysed in RIPA buffer containing protease inhibitors (Roche Life Science, Basel, Switzerland), and protein concentration was assessed using RcDc assay (BioRad, Marnes-la-Coquette, France). Western blot analysis was performed using 60 µg of protein from each sample separated on a 7% acrylamide gel. After transfer onto nitrocellulose membranes, CFTR was probed using antibody 660 (Cystic Fibrosis Foundation, Chapel Hill, NC, USA), and equal loading was assessed using anti-tubulin (SantaCruz, Dallas, TX, USA).

### 4.4. Halide-Sensitive Functional Assay

CFTR activity was measured in transiently transfected HEK293 cells using the halide-sensitive yellow fluorescent protein YFP-H148Q/I152L [57]. The day after transfection, cells were transferred to poly-L-lysine-coated 96-well black/clear bottom microplates. After 24 h, plates were washed with PBS, and each well was incubated for 30 min with 100 µL of PBS containing cpt-AMPc (100 µM) and IBMX (100 µM) (Sigma-Aldrich, Saint-Quentin-Fallavier, France). Plates were then transferred to a ClarioStar plate reader (BMG Labtech, Ortenberg, Germany) equipped with an injector, which enabled the continuous recording of fluorescence during the injection. After 5 s, 200 µL of PBS-NaI (PBS solution where NaCl is replaced with NaI) was injected.

### 4.5. Proximity Labeling

HEK293 cells were transiently transfected for 48 h in 10 cm diameter poly-L-lysine-coated dishes. Biotinylation was induced by adding biotin (Thermo Fisher Scientific, Illkirch-Graffenstaden, France) to the medium at 37 °C for 10 min (TurboID, 500 µM biotin) or 18 h (BioID, 50 µM biotin). For APEX2, the cells were pre-incubated for 30 min at 37 °C with Biotin-Phenol (500 μM, Iris Biotech, Marktredwitz, Germany), and the peroxidase activity was activated by the addition of H2O2 at a final concentration of 1 mM. The reaction was then quenched by the addition of quenching buffer (10 mM sodium azide, 10 mM sodium ascorbate and 5 mM Trolox, Sigma-Aldrich, Saint-Quentin-Fallavier, France). Cells were washed several times with PBS+ at 4 °C before being harvested and centrifuged. Lysis and streptavidin pull-down steps were performed, as previously described by Hung et al. [35].

### 4.6. NanoLC-MS/MS Protein Identification and Quantification

S-TrapTM micro spin column (Protifi, Farmingdale, NY, USA) digestion was performed on streptavidin eluates in 4× Laemmli buffer according to the manufacturer’s protocol but with 2 extra washing steps for thorough SDS elimination. Samples were digested with 2 µg of trypsin (Promega, Charbonnières-les-Bains, France) at 47 °C for 1 h 30 min. After elution, peptides were finally vacuum dried down and resuspended in 35 µL of 10% ACN and 0.1% TFA in HPLC-grade water prior to MS analysis. For each run, 5 µL was injected in a nanoRSLC-Q Exactive PLUS (RSLC Ultimate 3000) (Thermo Scientific, Illkirch-Graffenstaden, France). Peptides were loaded onto a µ-precolumn (Acclaim PepMap 100 C18, cartridge, 300 µm i.d. × 5 mm, 5 µm) (Thermo Scientific, Illkirch-Graffenstaden, France) and were separated on a 50 cm reversed-phase liquid chromatographic column (0.075 mm ID, Acclaim PepMap 100, C18, 2 µm) (Thermo Scientific, Illkirch-Graffenstaden, France). The chromatography solvents were (A) 0.1% formic acid in water and (B) 80% acetonitrile, 0.08% formic acid. Peptides were eluted from the column with the following gradients: 5% to 40% B (120 min), 40% to 80% (1 min). At 121 min, the gradient stayed at 80% for 5 min, and at 127 min, it returned to 5% to re-equilibrate the column for 20 min before the next injection. One blank was run between each series to prevent sample carryover. Peptides eluting from the column were analyzed by data-dependent MS/MS, using the top-10 acquisition method. Peptides were fragmented using higher-energy collisional dissociation (HCD). Briefly, the instrument settings were as follows: the resolution was set to 70,000 for MS scans and 17,500 for the data-dependent MS/MS scans in order to increase speed. The MS AGC target was set to 3.106 counts with a maximum injection time set to 200 ms, while the MS/MS AGC target was set to 1.105 with a maximum injection time set to 120 ms. The MS scan range was from 400 to 2000 m/z.

### 4.7. Data Processing Following LC-MS/MS Acquisition

The MS files were processed with the MaxQuant software version 2.0.1.0 and searched with the Andromeda search engine against the database of Homo sapiens from Swiss-Prot 04/2020. To search for parent mass and fragment ions, we set an initial mass deviation of 4.5 ppm and 20 ppm, respectively. The minimum peptide length was set to 7 amino acids, and strict specificity for trypsin cleavage was required, allowing up to two missed cleavage sites. Carbamidomethylation (Cys) was set as fixed modification, whereas oxidation (Met) and N-term acetylation were set as variable modifications. For APEX2, biotinylation (H23C18N3O3S) was set as variable modification on any tyrosine, tryptophane and histidine, and for TurboID, biotinylation (H14C10N2O2S) was set as variable modification on any lysine. A match between the runs was allowed. LFQ minimum ratio count was set to 2. The false discovery rates (FDRs) at the protein and peptide levels were set to 1%. Scores were calculated in MaxQuant, as described previously [58]. The reverse and common contaminants hits were removed from the MaxQuant output. Proteins were quantified according to the MaxQuant label-free algorithm using LFQ intensities [58,59]. Fragmentation visualization spectra were also extracted using the MQviewer integrated in the Maxquant software.

### 4.8. Data Processing and Statistical Analysis

Three to five independent experiments of HEK293 cells transfected with untagged CFTR, APEX2-CFTR-WT, TurboID-CFTR-WT, TurboID-CFTR-G551D, TurboID-CFTR-W1282X and KCNK3-TurboID were analyzed with Perseus software (version 1.6.15.0) freely available at www.perseus-framework.org [59]. The label-free quantification (LFQ) data were transformed in log2, and the Significance Analysis of INTeractome (SAINT [37]; https://reprint-apms.org/) was used for the identification of the proximal partners of CFTR on the raw MS files. Comparisons between CFTR mutants and the WT condition were performed with R software (version 4.1.0) based on the label-free quantification (LFQ) log2-transformed data. All proteins identified in all replicates of all conditions were subjected to Student’s *t*-test without correction for multiple testing. Where applicable, LogFC were shown as means, and *p*-values of less than 0.01 or 0.1 were considered. For CFTR comparison with KCNK3, the *p*-value was set at <0.01, since large differences were expected. For mutant comparison, since the differences were smaller, we lowered the stringency to a *p*-value of <0.1. The gene ontology enrichment calculations and lollipop graphs were generated with the ShinyGO v0.741 tool (http://bioinformatics.sdstate.edu/go74/ (accessed on February 2022)) [60].

## Data Availability

The mass spectrometry proteomics data were deposited to the ProteomeXchange Consortium via the PRIDE [61] partner repository with the dataset identifier PXD035184, and the protein interactions were submitted to the IMEx (http://www.imexconsortium.org) consortium through IntAct [62] and assigned the identifier IM-29540.

## Supplementary Materials

The following supporting information can be downloaded at: https://www.mdpi.com/article/10.3390/ijms23168937/s1.
