# Supplementary material for: Differential CFTR-Interactome Proximity Labeling Procedures Identify Enrichment in Multiple SLC Transporters
Source: Int J Mol Sci. 2022 Aug 11;23(16):8937. doi: 10.3390/ijms23168937 (PMC9408702; doi:10.3390/ijms23168937)
Supplement: Supplementary file 1 [file ijms-23-08937-s001.zip › Figures Supp+legend.pdf]

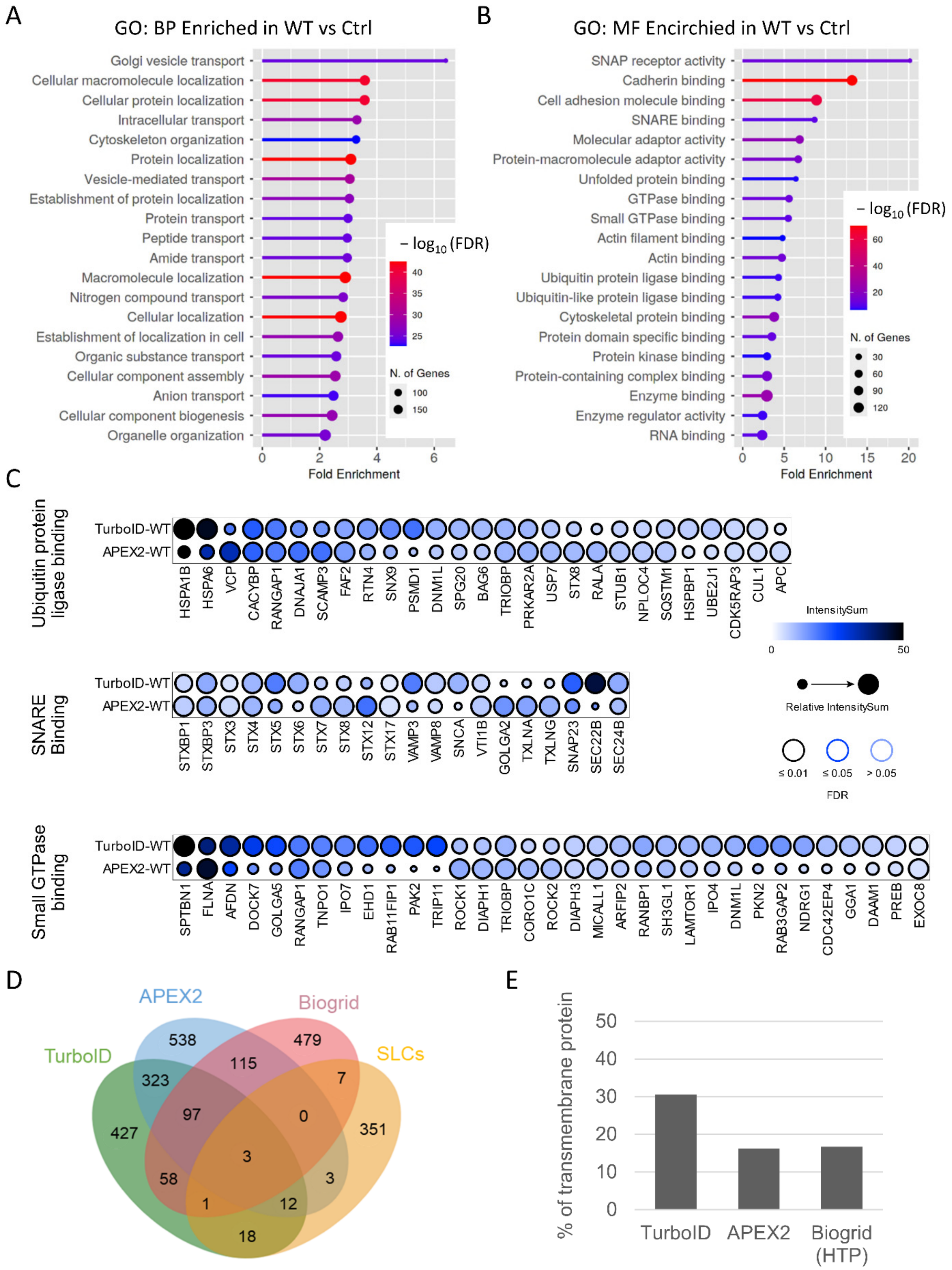

A
GO: BP Enriched in WT vs Ctrl
Golgi vesicle transport
Cellular macromolecule localization
Cellular protein localization
Intracellular transport
Cytoskeleton organization
Protein localization
Vesicle-mediated transport
Establishment of protein localization
Protein transport
Peptide transport
Amide transport
Macromolecule localization
Nitrogen compound transport
Cellular localization
Establishment of localization in cell
Organic substance transport
Cellular component assembly
Anion transport
Cellular component biogenesis
Organelle organization
Fold Enrichment
0 2 4 6
$-\log_{10}$(FDR)
40 35 30 25
N. of Genes
100
150
B
GO: MF Encirchied in WT vs Ctrl
SNAP receptor activity
Cadherin binding
Cell adhesion molecule binding
SNARE binding
Molecular adaptor activity
Protein-macromolecule adaptor activity
Unfolded protein binding
GTPase binding
Small GTPase binding
Actin filament binding
Actin binding
Ubiquitin protein ligase binding
Ubiquitin-like protein ligase binding
Cytoskeletal protein binding
Protein domain specific binding
Protein kinase binding
Protein-containing complex binding
Enzyme binding
Enzyme regulator activity
RNA binding
Fold Enrichment
0 5 10 15 20
$-\log_{10}$(FDR)
60 40 20
N. of Genes
30
60
90
120
C
Ubiquitin protein ligase binding
TurboID-WT
APEX2-WT
HSPA1B HSPA6 VCP CACYBP RANGAP1 DNAJA1 SCAMP3 FAF2 RTN4 SNX9 PSMD1 DNM1L SPG20 BAG6 TRIOBP PRKAR2A USP7 STX8 RALA STUB1 NPLOC4 SQSTM1 HSPBP1 UBE2J1 CDK5RAP3 CUL1 APC
IntensitySum
0 50
SNARE Binding
TurboID-WT
APEX2-WT
STXBP1 STXBP3 STX3 STX4 STX5 STX6 STX7 STX8 STX12 STX17 VAMP3 VAMP8 SNCA VTI1B GOLGA2 TXLNA TXLNG SNAP23 SEC22B SEC24B
Relative IntensitySum
≤ 0.01 ≤ 0.05 > 0.05
FDR
Small GTPase binding
TurboID-WT
APEX2-WT
SPTBN1 FLNA AFDN DOCK7 GOLGA5 RANGAP1 TNPO1 IPO7 EHD1 RAB11FIP1 PAK2 TRIP11 ROCK1 DIAPH1 TRIOBP CORO1C ROCK2 DIAPH3 MICALL1 ARFIP2 RANBP1 SH3GL1 LAMTOR1 IPO4 DNM1L PKN2 RAB3GAP2 NDRG1 CDC42EP4 GGA1 DAAM1 PREB EXOC8
D
APEX2
Biogrid
TurboID
SLCs
538
479
323
115
7
427
97
0
351
3
58
3
1
12
18
E
% of transmembrane protein
50
40
30
20
10
0
TurboID
APEX2
Biogrid (HTP)

Figure S1. Analysis CFTR enrichments

A-B. Gene ontology analysis of the 435 proteins identified with both APEX2 and TurboID procedures as high confident proximal partners (FDR<1%), using (**A)** Biological process (GO: BP) or (**B)** Molecular function (GO: MF). The strongest enrichments are seen for Golgi vesicle transport and SNAP receptor activity for Biological process and Molecular function, respectively. **C.** Selected Gene Ontology (Molecular Function) enriched in common APEX2 and TurboID high confident proximal partners (FDR<1%), shown as dot plots with ProHits-viz [42]. The color of each circle represents the intensity, the circle size indicates the relative value of the intensity across APEX2 and TurboID and confidence in the measurement via colored edge. **D.** Venn diagram performed between APEX2, TurboID, CFTR partners referenced in Biogrid database (either from low-throughput or high-throughput studies) and SLCs family proteins members. **E.** Bar graph representing the proportion in high confident interactors of transmembrane proteins in APEX2 and TurboID dataset and in the Biogrid database (HTP).

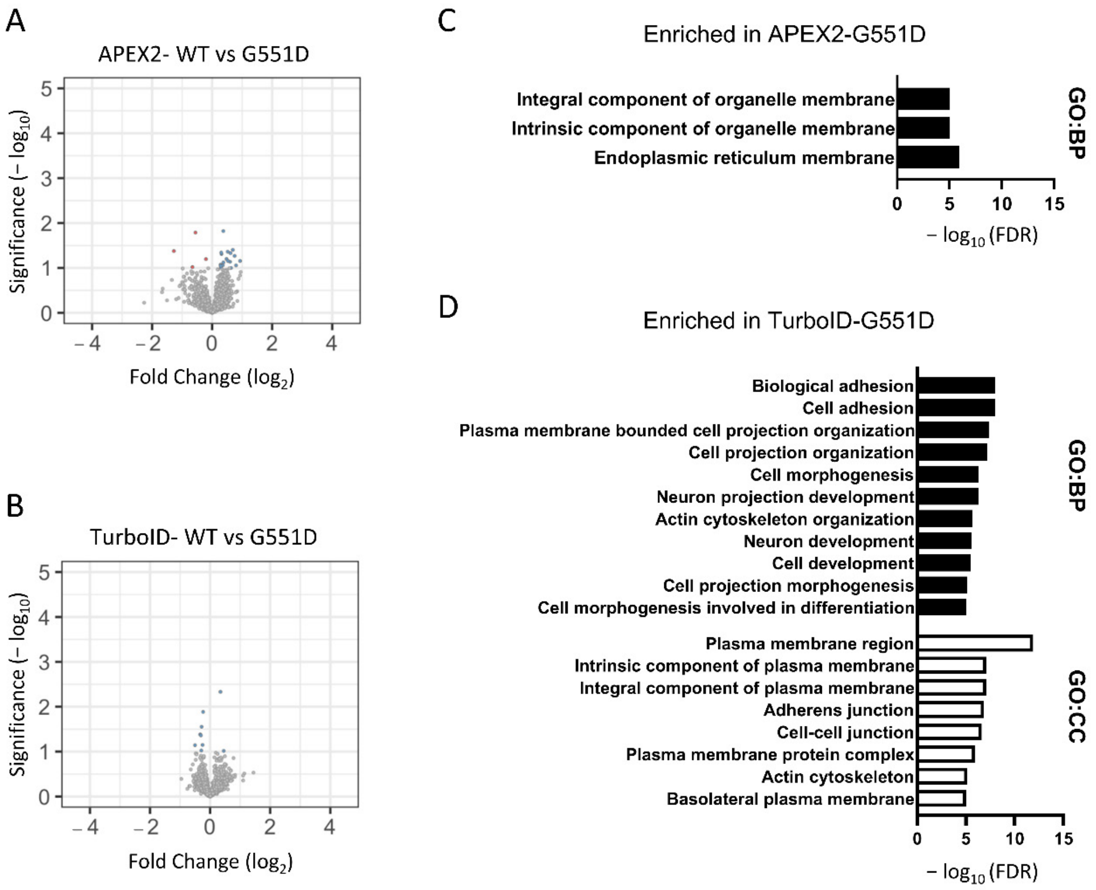

Figure S2. Analysis of CFTR-G551D and CFTR-W1282X proximal datasets.
A-B. Volcano plots of APEX2-CFTR-G551D (n=3, 1966 total proteins) (**A**) and TurboID-CFTR-G551D (n=4, 1654 total proteins) (**B**) versus WT-transfected HEK293 cells. Blue dots indicate proteins identified as enriched in the G551D sample and red dots in the WT sample. **C-D.** GO enrichment terms identified in APEX2-CFTR-G551D (**C**) and in TurboID-CFTR-G551D (**D**).

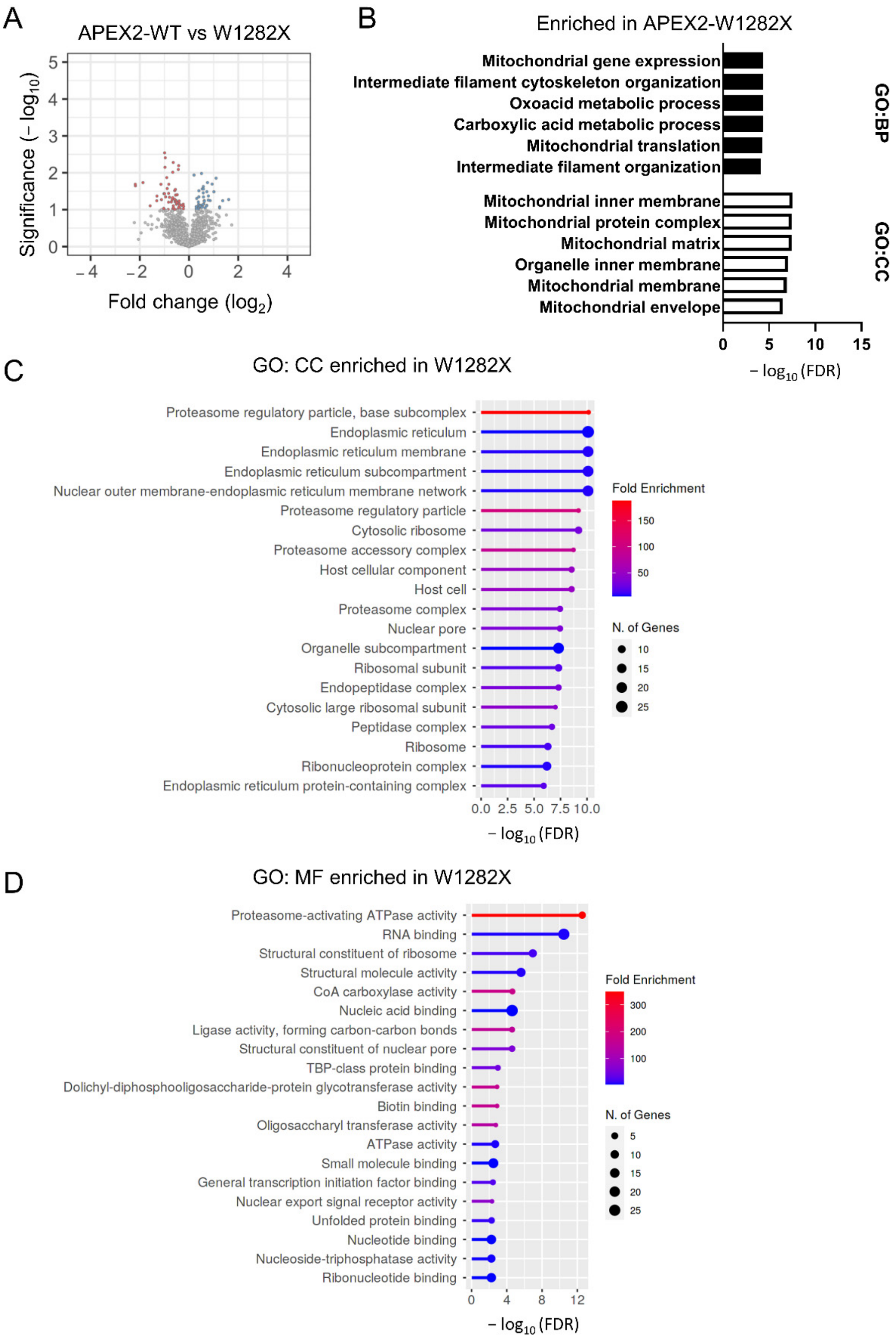

Figure S3. Analysis of TurboID-CFTR-W1282X proximal dataset

A. Volcano plot of APEX2-CFTR-W1282X (n=3, 1966 total proteins) versus APEX2-CFTR-WT transfected cells. Blue dots indicate proteins identified as enriched in the W1282X sample and red dots in the WT sample. **B.** GO enrichment terms identified in APEX2-CFTR-W1282X. **C-D.** GO enrichment terms identified in TurboID-CFTR-W1282X for cellular component (GO: CC) and Molecular Function (GO: MF).

A

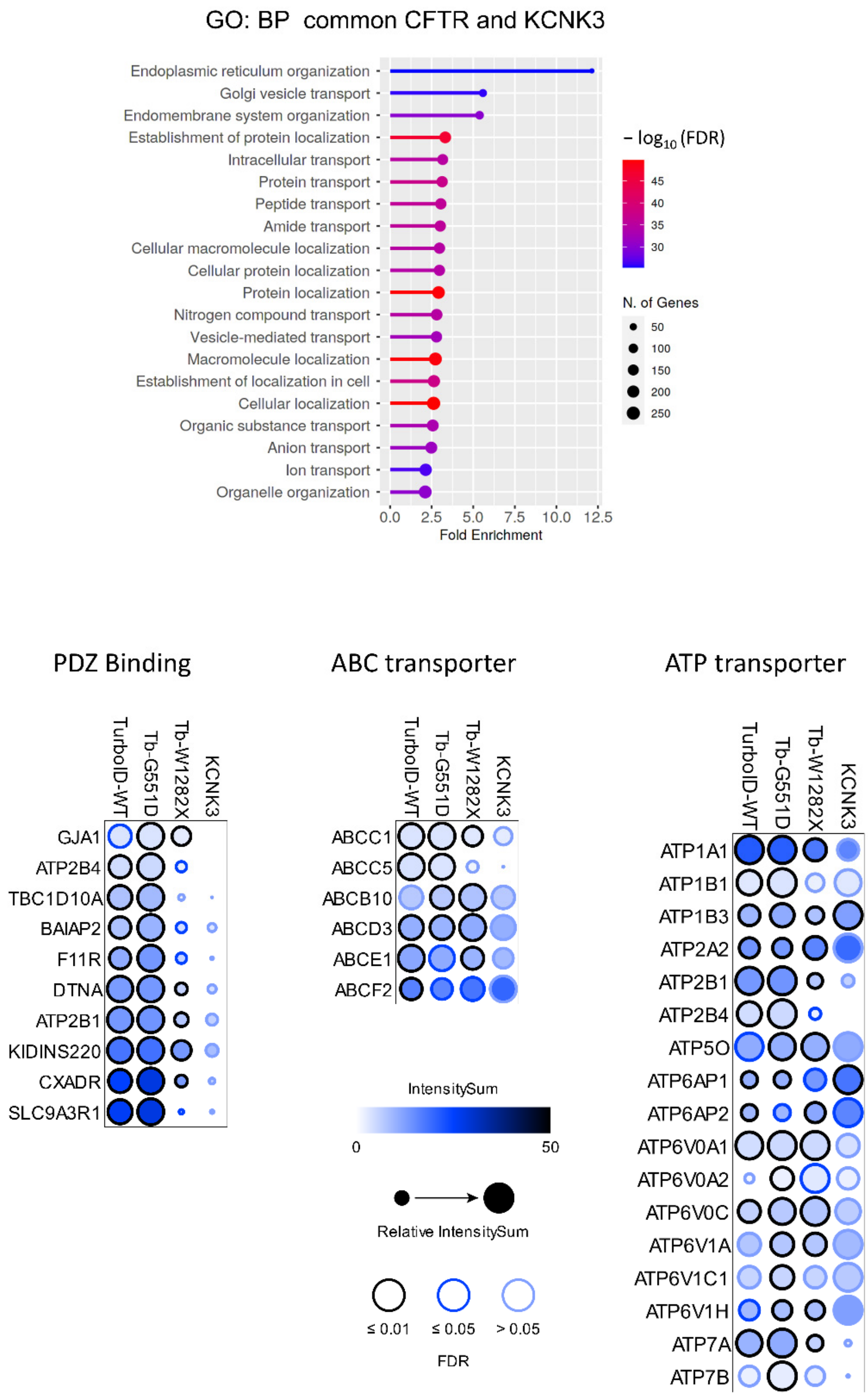

Figure S4. Analysis of CFTR and KCNK3 proximal datasets.
A. GO enrichment terms identified for common TurboID-CFTR and TurboID-KCNK3. **B.** ATP transporters, ABC transporters and PDZ Binding domain detected in at least one condition as High confident partners (FDR<1%) is shown as dot plots with ProHits-viz [42]. The color of each circle represents the intensity, the circle size indicates the relative value of the intensity across APEX2 and TurboID and confidence in the measurement via colored edge.

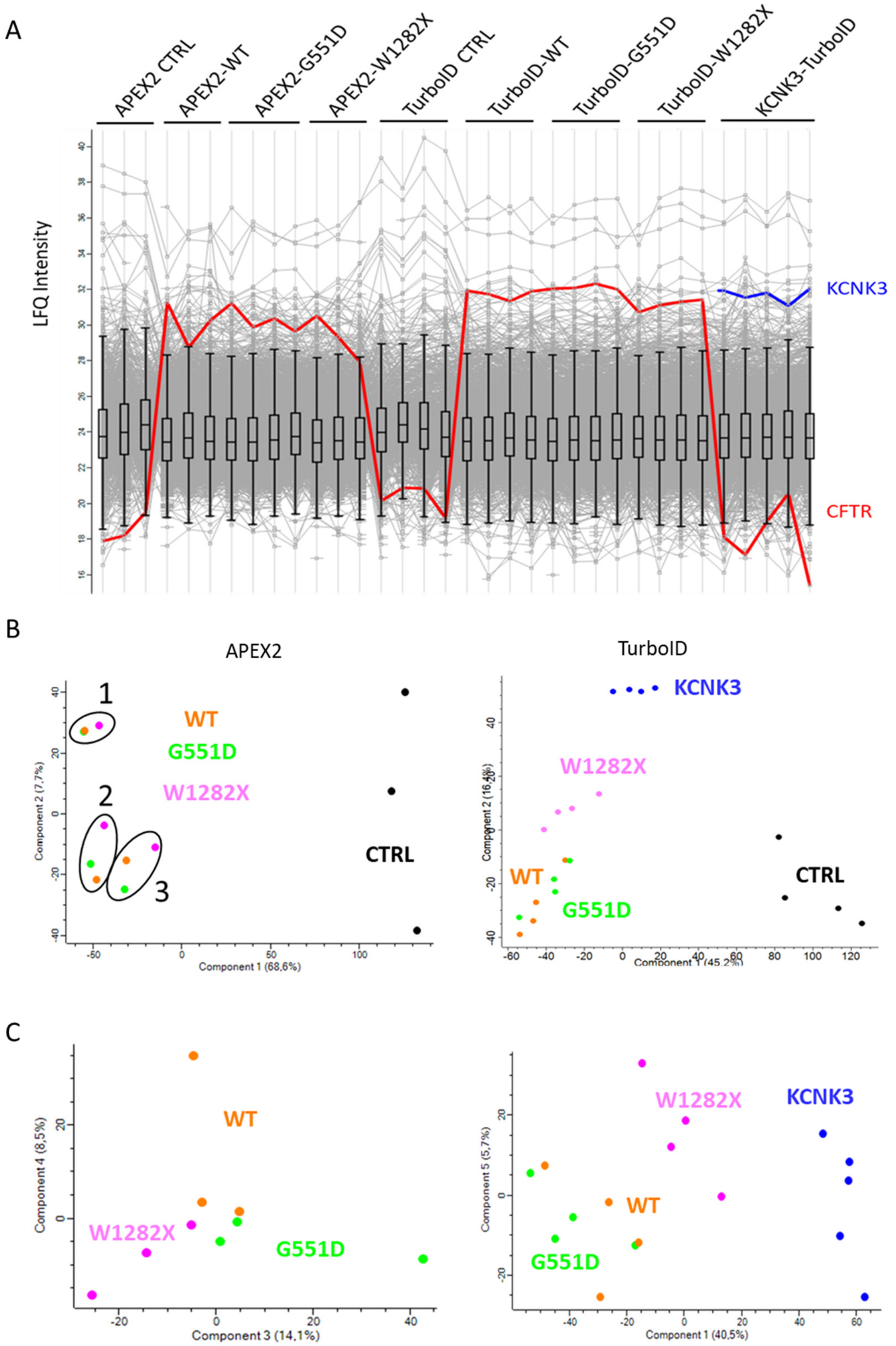

Figure S5. LFQ and PCA analysis of mass spectrometry data.

A. Label free quantitation (LFQ) of proteins identified by MS in control, APEX2 and TurboID conditions of CFTR-WT, CFTR-G551D, CFTR-W1282X and KCNK3 samples. CFTR LFQ intensity in each sample is indicated in red while KCNK3 LFQ intensity is in blue.

B-C. Principal component analysis (PCA) of APEX2 and TurboID datasets with (B) or without (C) the negative control condition. In B for APEX2, independent experiments are indicated by circle.

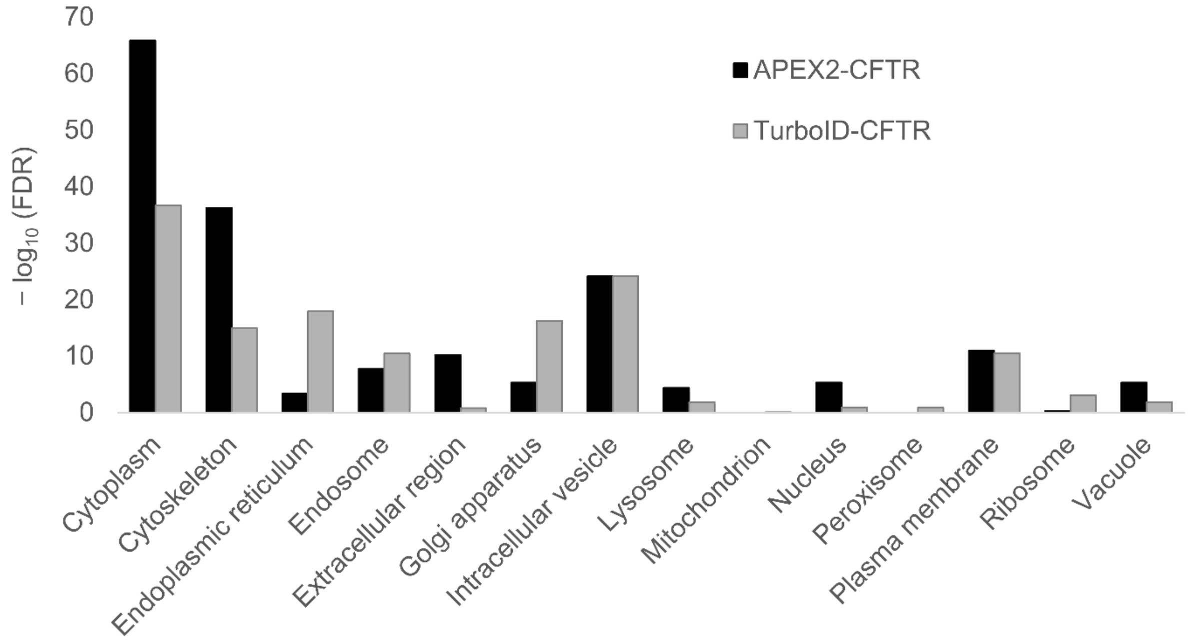

Figure S6. Subcellular distribution of the APEX2- and TurboID-WT data sets.
The subcellular distribution of the proteins identified using APEX2 (black bars) and TurboID (grey bars) was obtained using SubcellulaRVis which calculates enrichment for the 14 subcellular compartments indicated.
